# Calcium-Mediated Oscillation in Membrane Potentials and Atrial-Triggered Activity in Atrial Cells of Casq2^R33Q/R33Q^ Mutation Mice

**DOI:** 10.3389/fphys.2018.01447

**Published:** 2018-11-02

**Authors:** Jian-cheng Zhang, Hong-lin Wu, Qian Chen, Xiao-ting Xie, Tian Zou, Chao Zhu, Ying Dong, Guo-jian Xiang, Lei Ye, Yang Li, Peng-li Zhu

**Affiliations:** ^1^Department of Cardiology, Fujian Provincial Hospital, Provincial Clinical Medicine College of Fujian Medical University, Fuzhou, China; ^2^Department of Critical Care Medicine Division Four, Fujian Provincial Hospital, Provincial Clinical Medicine College of Fujian Medical University, Fuzhou, China; ^3^Department of Cardiology, General Hospital of People’s Liberation Army, Beijing, China; ^4^National Heart Research Institute Singapore, National Heart Centre Singapore, Singapore, Singapore; ^5^Department of Geriatric Medicine, Fujian Provincial Center for Geriatrics, Fujian Provincial Hospital, Provincial Clinical Medicine College of Fujian Medical University, Fuzhou, China

**Keywords:** Ca^2+^-triggered activity, AF, SCaE, oscillation in membrane potentials, EADs/DADs, ITi, CaMKII

## Abstract

**Aim:** We investigated the underlying mechanisms in atrial fibrillation (AF) associated with R33Q mutation and Ca^2+^-triggered activity.

**Methods and Results:** We examined AF susceptibility with intraesophageal burst pacing in the sarcoplasmic reticulum (SR) Ca^2+^ leak model calsequestrin 2 R33Q (Casq2^R33Q/R33Q^) mice. Atrial trigger appeared in R33Q mice but not WT mice (17.24%, 5/29 vs. 0.00%, 0/32, *P <* 0.05). AF was induced by 25 Hz pacing in R33Q mice (48.27%, 14/29 vs. 6.25%, 2/32, *P <* 0.01). The mice were given 1.5 mg/kg isoproterenol (Iso), and the incidences of AF increased (65.51%, 19/29 vs. 9.21%, 3/32, *P <* 0.01). Electrophysiology experiments and the recording of intracellular Ca^2+^ indicated significant increases in the Ca^2+^ sparks (5.24 ± 0.75 100 μM^-1^.s^-1^ vs. 0.29 ± 0.04 100 μM^-1^.s^-1^, *n* = 20, *P <* 0.05), intracellular free Ca^2+^ (0.238 ± 0.009 μM vs. 0.172 ± 0.006 μM, *n* = 20, *P <* 0.05), Ca^2+^ wave (11.74% vs. 2.24%, *n* = 20, *P <* 0.05), transient inward current (ITi) (-0.56 ± 0.02 pA/pF vs. -0.42 ± 0.01 pA/pF, *n* = 10, *P <* 0.05), and oscillation in membrane potentials (10.71%, 3/28 vs. 4.16%, 1/24, *P <* 0.05) in the R33Q group, but there was no significant difference in the L-type calcium current. These effects were enhanced by Iso, and the inhibition of calmodulin-dependent protein kinase II (CaMKII) by 1 μM KN93 reversed the effects of Iso on Ca^2+^ sparks (5.01 ± 0.66 100 μm^-1^.s^-1^ vs. 11.33 ± 1.63 100 μm^-1^.s^-1^, *P* < 0.05), intracellular Ca^2+^ (0.245 ± 0.005 μM vs. 0.324 ± 0.008 μM, *P* < 0.05), Ca^2+^ wave (12.35% vs. 17.83%, *P <* 0.05), ITi (-0.61 ± 0.02 pA/pF vs. -0.78 ± 0.03 pA/pF, *n* = 10, *P <* 0.05), and oscillation in membrane potential (17.85% 5/28 vs. 32.17% 9/28, *P <* 0.05). The reduction of ryanodine receptor 2 (RyR2) stable subunits (Casq2, triadin, and junctin) rather than RYR2 and the increase in CaMKII, phosphor-CaMKII, phosphor-RyR2 (Ser 2814), SERCA, and NCX1.1 was reflected in the R33Q group.

**Conclusion:** This study demonstrates that the increase in spontaneous calcium elevations corresponding to ITi that may trigger the oscillation in membrane potentials in the R33Q group, thereby increasing the risk of AF. The occurrence of spontaneous calcium elevations in R33Q atrial myocytes is due to the dysfunction of RyR2 stable subunits, CaMKII hyperactivity, and CaMKII-mediated RyR phosphorylation. An effective therapeutic strategy to intervene in Ca^2+^-induced AF associated with the R33Q mutation may be through CaMKII inhibition.

## Introduction

Atrial fibrillation (AF) is one of the most common arrhythmias. In a canine atrial tachyarrhythmia model, it was reported that intracellular Ca^2+^ overload occurs during AF, and that the intracellular Ca^2+^ concentration subsequently returns to normal after AF (Akar et al., 2003). The increased sensitivity of Ca^2+^ release channels indicates the preformation of spontaneous calcium elevations (SCaE), consisting of Ca^2+^ sparks and Ca^2+^ waves associated with a high level of intracellular Ca^2+^ content (Wier et al., 1987).

It is well known that Ca^2+^ is mainly released from the sarcoplasmic reticulum (SR) by the ryanodine receptor 2 (RyR2) channel, but the number of RyR2 channels in patients with chronic AF is significantly reduced or unchanged (Tang et al., 2007; Greiser and Schotten, 2013), and it is difficult to explain why SCaEs increase in AF. It was reported that “hyperphosphorylation” of RyR2 appears to be the main factor responsible for unstable channels (Voigt et al., 2012). However, RYR2 phosphorylation was unaltered in a patient with paroxysmal AF, whereas the RYR2 single-channel open probability was increased (Voigt et al., 2014). The RyR2 stable subunits include calsequestrin 2 (Casq2), triadin, and junctin, all of which are involved in the regulation of Ca^2+^ release (Franzini-Armstrong et al., 2005). Most AF cases attach emphasis to the amount and function of RYR2, but <5% of cases are focused on RYR2 stable subunits, and research exploring the link between Casq2 and AF has rarely been conducted. The loss of Casq2, as a free Ca^2+^ buffer in the SR (Gaburjakova et al., 2013), resulted in bradycardia, sinoatrial node conduction abnormalities, and beat-to-beat heart rate variability in a study conducted by Glukhov et al. (2015), and Faggioni et al. (2014) found that there is an increased risk of AF in Casq2^-/-^ mice. Casq2-R33Q mice display an adaptive pattern, and the sensitivity of RyR2s from Casq2-R33Q mice was between that of Casq2^-/-^ and WT mice (Valle et al., 2014). Few studies have been performed that examine the association of Casq2-R33Q and AF, and whether there is the same action on AF with Casq2^-/-^ and Casq2-R33Q mutations is uncertain, given the lack of support of experiments.

Membrane electrophysiological activities refer to afterdepolarizations, which are due to early afterdepolarizations (EADs) or delayed afterdepolarizations (DADs). It is well known that afterdepolarizations are responsible for atrial-triggered activity and are associated with various Ca^2+^-sensitive currents (Sipido et al., 1995; Lo et al., 2013). The L-type calcium current (ICa-L) prolongs the action potential duration and initiates phase 2 EADs during action potential (Madhvani et al., 2015), whereas the Na–Ca exchange current (INCX) activates the transient inward current (ITi) and initiates DADs (Kao et al., 2010). More recently, the relationship has been explored between intracellular Ca^2+^ oscillations and the oscillation in membrane potentials in the development of AF. Faggioni et al. (2014) discovered that AF was associated with DADs induced by a Ca^2+^ leak. Moreover, EADs also play a predominant role in Ca^2+^-trigged activity. Few studies have been performed that explored the abnormalities in cellular electrophysiology, channel currents, and intracellular calcium ions of atrial myocytes in Casq2-R33Q mice. Our goal in this study was to elucidate the changes in the cellular electrophysiology and the channel current in atrial myocytes in Casq2-R33Q mice.

## Study Design

Casq2^R33Q/R33Q^ mice have mutated Casq2 genes that provide an SR Ca^2+^ leak model for this study. Firstly, we organized an *in vivo* study to check the AF susceptibility in this animal model with an SR Ca^2+^ leak. Secondly, we planned to demonstrate that Ca^2+^ changes were associated with the changes in Ca^2+^-sensitive currents and the oscillation in membrane potentials. Finally, we investigated the effects of interventions including the Iso and calmodulin-dependent protein kinase II (CaMKII) on Ca^2+^ levels, Ca^2+^ sensitive currents, and oscillation in membrane potentials.

## Materials and Methods

### Animal Model Generation

The Casq2^R33Q/R33Q^ mouse model was provided by Dr. Nicoletta Rizzi (Molecular Cardiology, Pavia, Italy), and the genotype characteristics were determined. Mouse housing and care were provided by PLA General Hospital Laboratories (Beijing, China). All our experimental procedures were approved by the Ethics Committee of PLA General Hospital and performed in accordance with the Guide for the Care and Use of Laboratory Animals published by the U.S. National Institutes of Health (Publication No. 23, revised 1996).

### Electrophysiology Study

Surface II-lead ECG was used to obtain ECG recordings in the *in vivo* study. Mice were ECG-monitored for 6 h in our observation unit, and a premature atrial trigger was defined as >3 consecutive supraventricular beats. Up to 3 s, 25 Hz intraesophageal burst pacing was applied to assess the susceptibility to AF. AF was defined as an irregular atrial tachycardia (>800 bpm) lasting for at least 5 s immediately after a burst pacing cycle. After the mice were given 1.5 mg/kg Iso by intraperitoneal injection, the induction of AF was recorded again at 25 Hz atrial burst pacing.

### Single Atrial Myocyte Preparation

The Langendorff system was used to prepare single atrial myocytes. The hearts of mice were perfused in Ca^2+^-free Tyrode’s solution immediately after removal. The Ca^2+^-free Tyrode solution with pH of 7.40 ± 0.05 (nmol/L) was composed of NaCl 113, KCl 4.7, KH_2_PO_4_ 0.6, Na_2_HPO_4_ 0.6, MgSO_4_ 1.2, NaHCO_3_ 12, KHCO_3_ 12, HEPES 10, taurine 30, glucose 5, and BDM 10. First, isolated hearts were perfused for 4–5 min with Tyrode buffer solution. Then, isolated hearts were perfused for 11–14 min with Tyrode’s solution containing 1 mg/ml collagen II and 0.25 mg/ml trypsin. The left atria were removed and cut into pieces in enzyme solution to single atrial cells. Then, isolated atrial cells were stored in low Ca^2+^ (0.5 mM) Tyrode’s solution. Atrial myocytes were moved into a 1.8 mM Ca^2+^-containing Tyrode’s solution for measuring intracellular Ca^2+^ and membrane currents/potentials.

### Confocal Ca^2+^ Imaging and Intracellular Ca^2+^ Quantification

Confocal Ca^2+^ imaging was used to detect intracellular Ca^2+^ changes by recording Ca^2+^ sparks and Ca^2+^ waves as well as the times to Ca^2+^ peak and Ca^2+^ decay. Fluo-4 acetoxymethyl (AM) ester dye (Thermo Fisher Scientific) is a non-ratiometric Ca^2+^ indicator that is commonly used with 488 nm excitation. Fluo-4-loaded atrial myocytes were exposed in different solutions including a control solution, solution with 1 μM Iso (Sigma), or solution with both 1 μM KN92 (Sigma) and 1 μM KN93 (Sigma) to assess the different effects of these solutions on Ca^2+^ dynamics. After de-esterification, a laser-scanning confocal microscope (SP5, Leica Microsystems) was used to record spontaneous Ca^2+^ release events (SCaEs) and the constant rate of Ca^2+^ cycling under different conditions.

The atrial myocyte intracellular Ca^2+^ level was quantified with Fura-2 (a ratiometric Ca^2+^ indicator) (Kao et al., 2010). A [Ca^2+^]i trace was derived from the ratio trace by using the following calibration calculation formula: [Ca^2+^]i = Kd(R-Rmin)(s_f,2_/s_b,2_)/(Rmax–R). A different fluorescence intensity was observed for Fura-2 at 340 and 380 nm wavelength (F340 and F380), and *R* = F340/F380, where Rmin is the value of *R* in the Ca^2+^-free solution, Rmax is the value of R when the indicator is saturated with Ca^2+^, and s_f,2_/s_b,2_ is simply the ratio of the measured fluorescence intensity in Ca^2+^-free and Ca^2+^-bound solutions; *K*d = 224 nM. To measure spontaneous Ca^2+^ waves, the atrial cells were paced at 1 Hz for 10 s, and then were monitored for 10 s. After the 1 Hz pacing stimulation stopped, the external solution was rapidly switched to a free Na^+^ and Ca^2+^ solution to inhibit Ca^2+^ transportation by NCX; this was followed by 10 s of monitoring to record the Ca^2+^ spark frequency and another 10 s of monitoring to record the intracellular baseline Ca^2+^ (Sacherer et al., 2012).

### Electrophysiology Experiments

Electrophysiology experiments were used to study the relationship between ICa-L or ITi and the action potential. Currents (ICa-L and ITi) and action potentials were recorded using the whole-cell patch-clamp technique with an Axon-700B amplifier (Axon Instruments). The action potentials and the action potential amplitudes were measured under the current clamp mode with 2.5 ms/1 nA depolarizing pulses. The whole-cell currents including ICa-L and ITi were obtained under the voltage clamp mode. ICa-L was recorded with a 10 mV voltage step from a holding potential of -40 mV to +70 mV. ITi was recorded with an increment of 10 mV voltage potential from -100 mV to +10 mV. These recordings were used to create the current–voltage relationship, steady state activation (SSA), or steady-state inactivation (SSI) curves (SSA left shift indicates the activation of ICa-L and SSI right shift indicates the inactivation of ICa-L). The electrophysiology data were analyzed by Clampfit version 10.4 (Axon Instruments) and Origin (Microcal Software). Triggered activity was defined as an unstimulated action potential arising from a DAD or an EAD.

### Western Blot Analysis

Western blot was used to investigate changes in the SR membrane calcium channel receptor RyR2 and its subunits. Total protein was obtained from 12-week-old Casq2^R33Q/R33Q^ mice and WT mice. Protein samples were separated on 4–8 and 12–15% gels using sodium dodecyl sulfate-polyacrylamide gel electrophoresis (SDS-PAGE) and then transferred onto nitrocellulose membranes. Western blot was performed under one of the unique antibodies including anti-RyR2 (abcam), anti-phospho-RyR2 (Badrilla), anti-CaMKII (abcam), anti-phospho-CaMKII (abcam), anti-CASQ (ABR), anti-triadin (ABR), anti-junctin (ABR), anti-NCX1.1 (abcam), and anti-SERCA (abcam), and then incubated overnight at 4°C for specialty binding. Chemiluminescent detection was performed with substrate reagents (Solarbio, Germany). Densitometric analysis of Western blot data was performed using ImageJ software.

### Statistical Analysis

Statistical analysis was performed using SPSS v.17 software. The data are expressed as the mean ± SD. One-way ANOVA with Bonferroni *post hoc* analysis or student’s *t*-test was used for comparison between groups. Fisher’s exact test was used to compare the incidences of atrial tachyarrhythmia and oscillation in membrane potentials (DADs/EADs) between the groups. A *P*-value less than 0.05 was considered statistically significant.

## Results

### Increased AF Susceptibility in Casq2^R33Q/R33Q^ Mice

**FIGURE 1:**
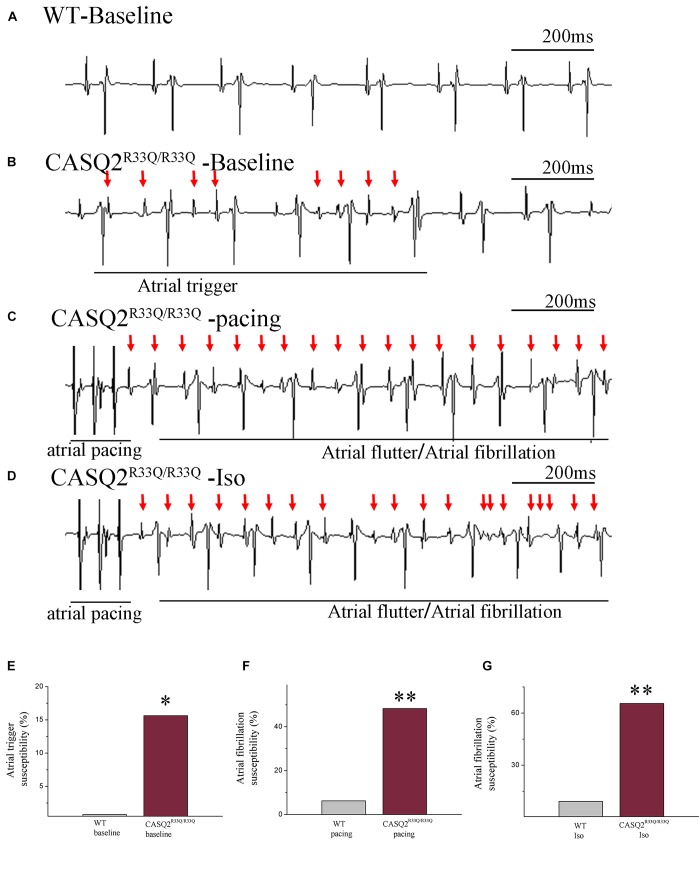
Casq2^R33Q/R33Q^ hearts are susceptible to atrial tachyarrhythmia, indicated by the **(A)** ECG record in the Casq2^R33Q/R33Q^ mice compared with sinus rhythm in the WT mice under intraesophageal pacing at 25 Hz. **(B)** Atrial tachyarrhythmia resembles atrial trigger, and **(C,D)** atrial flutter and AF were observed in Casq2^R33Q/R33Q^ hearts. The rate of atrial tachyarrhythmia susceptibility under **(E)** baseline status, **(F)** pacing condition, and **(G)** Iso stimulation in each group. **(F)** The probability of AF induction at 25 Hz burst pacing in the Casq2^R33Q/R33Q^ mice and the WT mice given 1.5 mg/kg Iso. ^∗^*P* < 0.05 vs. WT, ^∗∗^*P* < 0.01 vs. WT.

We first determined the atrial-triggered activity susceptibility *in vivo* at the baseline; this showed that the atrial-triggered activity appears in 5 of 29 (17.24%) Casq2^R33Q/R33Q^ mutation mice in contrast to 0 of 32 (0.00%) WT mice (*P <* 0.05). The episodes of AF and atrial flutter were labeled together as AF, and AF was induced by 25 Hz burst pacing in 14 of 29 (48.27%) Casq2^R33Q/R33Q^ mutation mice in contrast to 2 of 32 (6.25%) WT mice (*P <* 0.01). After an intraperitoneal injection of 1.5 mg/kg Iso, the incidences of AF were increased to 19 of 29 (65.51%) Casq2^R33Q/R33Q^ mutation mice compared with 3 of 32 (9.21%) WT mice (*P <* 0.01) (Figure 1).

### Increased SCaE and Basal Ca^2+^ Level in Casq2^R33Q/R33Q^ Atrial Myocytes

**FIGURE 2:**
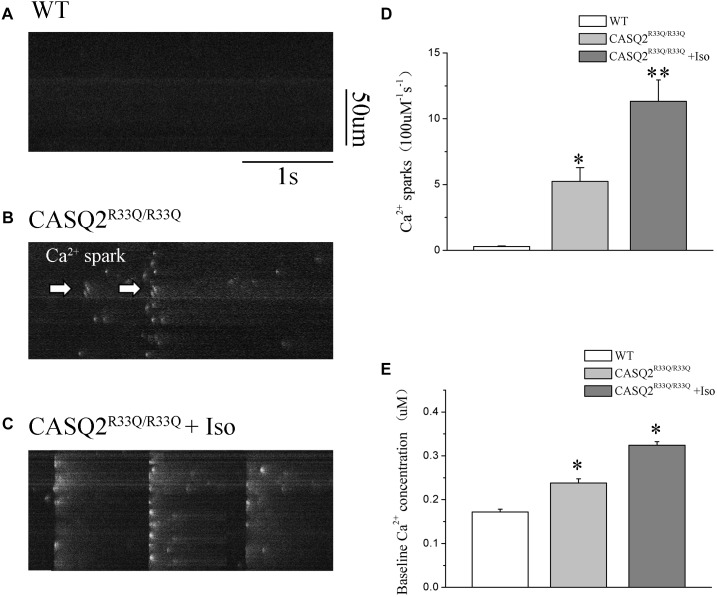
**(A–C)** Original recordings of Ca^2+^ spark in the WT and Casq2^R33Q/R33Q^ atrial myocytes, as well as Casq2^R33Q/R33Q^ atrial myocytes with 1 μM isoproterenol. **(D)** Spontaneous Ca^2+^ spark reflected by the incidence of Ca^2+^ spark frequency (100 μm^-1^.s^-1^). **(E)** Comparison baseline Ca^2+^ concentration among groups. ^∗^*P* < 0.05 vs. WT, ^∗∗^*P* < 0.01 vs. WT.

**FIGURE 3:**
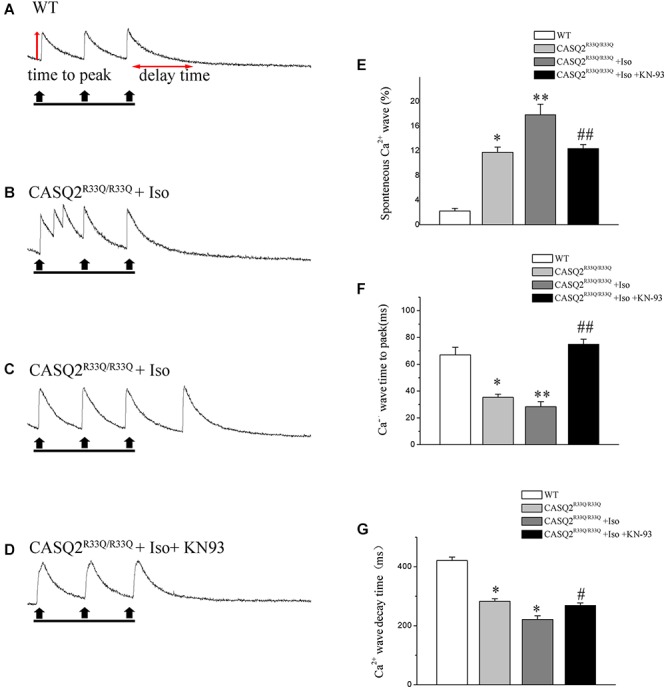
**(A)** Original recordings of spontaneous Ca^2+^ wave in the WT and Casq2^R33Q/R33Q^ atrial myocytes, as well as Casq2^R33Q/R33Q^ atrial myocytes with 1 μM isoproterenol. **(B)** A line record of Ca^2+^ waves that elicit EADs during an long AP and **(C)** a DAD after a regular AP. **(E–G)** Spontaneous Ca^2+^ release events reflected by the incidence of reactive Ca^2+^ wave (%), Ca^2+^ wave time to peak (ms), and Ca^2+^ wave decay time (ms) on each group. ^∗^*P* < 0.05 vs. WT, ^∗∗^*P* < 0.01 vs. WT. ^#^*P* < 0.05 and ^##^*P* < 0.01 when comparing the Iso group with the Iso plus KN93 group.

Basal intracellular Ca^2+^ levels were measured under no pacing or other interventions. Our study showed that the baseline intracellular Ca^2+^ level was higher in the R33Q mutation atrial myocytes compared with the WT atrial myocytes (0.238 ± 0.009 μM vs. 0.172 ± 0.006 μM, *n* = 20, *P <* 0.05) (Figure 2). The R33Q mutation atrial myocytes exhibited higher Ca^2+^ sparks (5.24 ± 0.75 100 μM^-1^.s^-1^ vs. 0.29 ± 0.04 100 μM^-1^.s^-1^, *n* = 20, *P <* 0.05) and spontaneous Ca^2+^ waves (11.74 vs. 2.24%, *n* = 20, *P <* 0.05) as compared to WT atrial myocytes. After treatment with 1 μM Iso, Ca^2+^ waves (17.83 vs. 11.74%, *n* = 20, *P <* 0.01), Ca^2+^ sparks (11.33 ± 1.63 100 μM^-1^.s^-1^ vs. 5.24 ± 1.05 100 μM^-1^.s^-1^, *n* = 20, *P <* 0.05), and intracellular Ca^2+^ (0.324 ± 0.008 μM vs. 0.238 ± 0.009 μM, *n* = 20, *P <* 0.05) were significantly increased only in the R33Q group. Furthermore, the time to Ca^2+^ peak was shortened by Iso (30.37 ± 4.36 ms vs. 37.64 ± 3.64 ms, *n* = 20, *P <* 0.05), and the decay time of Ca^2+^ waves (reflecting the time of Ca^2+^ restoration by SR) was significantly lowered by Iso (221.56 ± 9.44 ms vs. 401.17 ± 6.64 ms, respectively, *n* = 20, *P <* 0.01) in the R33Q group compared with the WT group (Figure 3).

**FIGURE 4:**
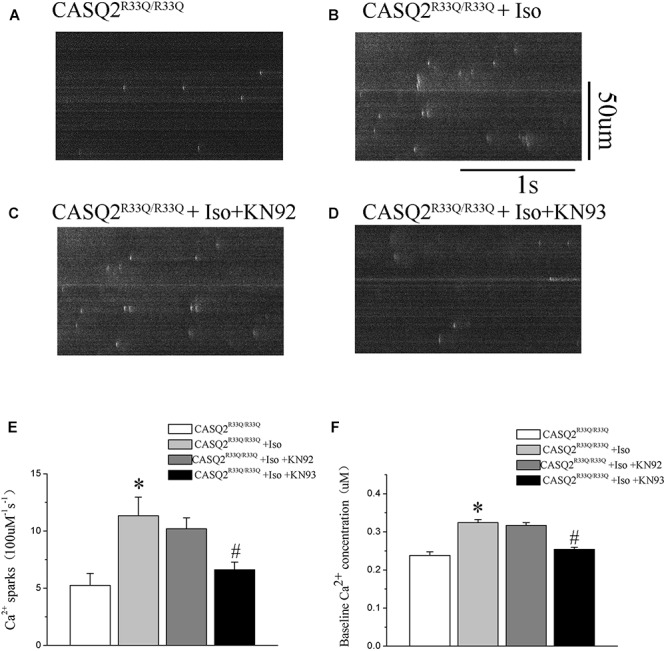
**(A)** The occurrence of EAD and DAD in the Casq2^R33Q/R33Q^ group by recording AP. **(B)** DADs (red arrow) from the mutated atrial myocytes. **(C)** EADs (blue arrows) from mutated atrial myocytes. **(D)** Effects of CaMKII inhibition with KN93 (1 μM) on membrane oscillations in Casq2^R33Q/R33Q^ myocytes. DADs/EADs were defined as membrane depolarization more than 20 mV. **(E)** Proportion of cells with EADs or DADs at 1 Hz in the Casq2^R33Q/R33Q^ and the WT groups with or without Iso. **(F)** Resting membrane potential (RMP) with Iso or without Iso between the two groups, and with Iso plus KN93 in the R33Q group. Each point/bar represents the mean ± SEM. ^∗^*P* < 0.05 vs. WT. ^#^*P* < 0.05 when comparing the Iso group with the Iso plus KN93 group.

Calmodulin-dependent protein kinase II is one of the main signaling pathways of β-adrenergic stimulation. We assessed the effect of the CaMKII inhibitor (KN93) on the Iso-induced-SCaE in the Casq2^R33Q/R33Q^ atrial myocytes. After the Casq2^R33Q/R33Q^ atrial myocytes were treated with 1 μM KN93, the Ca^2+^ waves (12.35 vs. 17.83%, *n* = 20, *P <* 0.05), Ca^2+^ sparks (5.01 ± 0.66 100 μM^-1^.s^-1^ vs. 11.33 ± 1.63 100 μM^-1^.s^-1^, *n* = 20, *P <* 0.05), and intracellular baseline Ca^2+^ (0.245 ± 0.005 μM vs. 0.324 ± 0.008 μM, *n* = 20, *P <* 0.05) were significantly reduced. KN-92 was used as a control compound to elucidate the inactivity of KN-93. Casq2^R33Q/R33Q^ atrial myocytes with 1 μM KN92 still developed Ca^2+^ sparks at a higher level (10.19 ± 0.96 100 μM^-1^.s^-1^ vs. 11.33 ± 1.63 100 μM^-1^.s^-1^, *n* = 20, *P* < 0.05) as well as a higher intracellular baseline Ca^2+^ (0.251 ± 0.007 μM vs. 0.324 ± 0.008 μM, *n* = 20, *P* < 0.05) (Figure 4).

### Changes in ICa-L and ITi in the Casq2^R33Q/R33Q^ Atrial Myocytes

**FIGURE 5:**
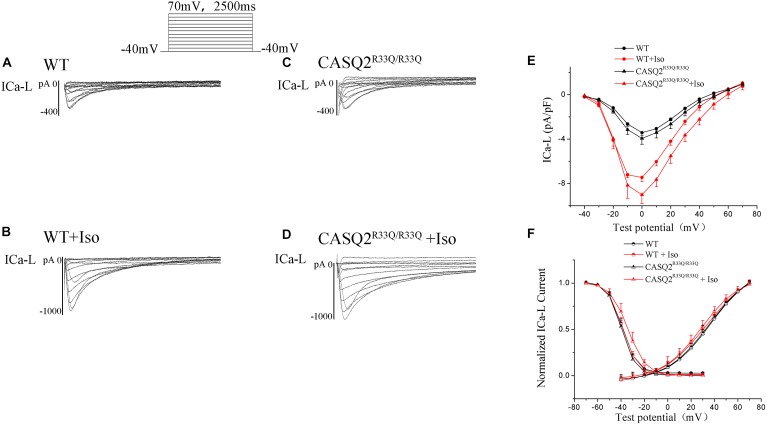
**(A–D)** The ICa-L in the Casq2^R33Q/R33Q^ and WT groups with or without Iso. ICa-L current traces in 10 mV increments from a holding potential of –40 mV to +70 mV in the WT and the Casq2^R33Q/R33Q^ atrial myocyte groups. **(E)** The current–voltage relation curve (I–V curve) for ICa-L. **(F)** SSA and SSI for ICa-L in the Casq2^R33Q/R33Q^ and the WT groups with and without Iso.

There was no significant difference in the ICa-L peak between the Casq2^R33Q/R33Q^ and WT groups (-3.94 ± 0.53 pA/pF vs. -3.42 ± 0.26 pA/pF, *n* = 10, *P* > 0.05), and ICa-L was increased in both groups by 1 μM Iso, with a more significant increase in the Casq2^R33Q/R33Q^ group (PA/PF: -9.01 ± 0.78 in the Casq2^R33Q/R33Q^ group vs. -7.45 ± 0.37 in the WT group, *n* = 10, *P <* 0.05). There was no significant difference in the SSA curve of ICa-L but a slight shift to the right in the SSI curve by Iso in the R33Q mutation group (SSI V_1/2_: from -39.54 to -34.05 mV vs. from -38.97 to -37.57 mV in the WT group, *n* = 10, *P <* 0.05), indicating that ICa-L was increased by Iso without a significant change in the L-type calcium channel function in the R33Q mutation group (Figure 5).

**FIGURE 6:**
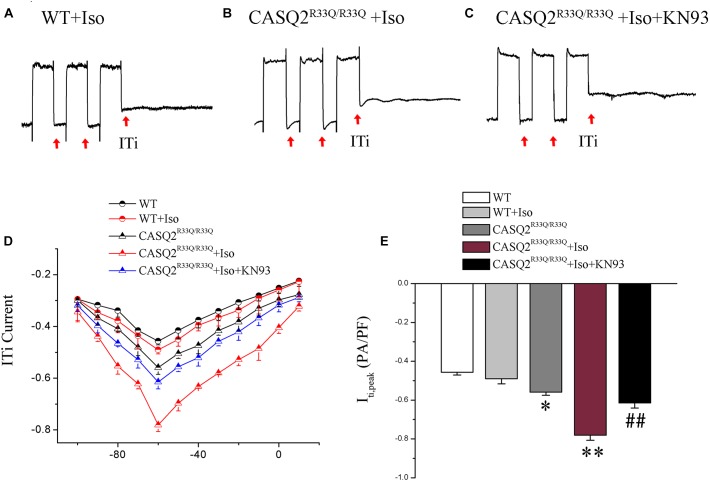
**(A–C)** ITi current traces from the WT and Casq2^R33Q/R33Q^ atrial myocytes. ITi currents recorded at various membrane potentials from –100 to +10 mV after repeated repolarizing trains from –80 to +50 mV. **(D)** The current–voltage relation curve (I–V curve) for ITi in two groups of atrial myocytes with and without Iso. **(E)** Comparison of ITi_peak_ current among groups of atrial myocytes. ^∗^*P* < 0.05 vs. WT, ^∗∗^*P* < 0.01 vs. WT. ^##^*P* < 0.01 when comparing the Iso group with the Iso plus KN93 group.

Transient inward current (ITi) is the main component of Na–Ca exchange current (INCX). We found that there was a significant increase in the ITi density (-0.56 ± 0.017 pA/pF vs. -0.42 ± 0.014 pA/pF, *n* = 10, *P* < 0.05) and a dramatic increase in ITi after exposure to Iso (-0.78 ± 0.03 pA/pF vs. -0.45 ± 0.02, *n* = 10, *P* < 0.01) in the R33Q group compared with the WT group. However, the effects of Iso were reversed by 1 μM KN93 (from -0.78 ± 0.03 pA/pF to -0.61 ± 0.02 pA/pF, *n* = 10, *P <* 0.05) (Figure 6).

### Increased Oscillation in Membrane Potentials in Atrial Myocytes of Casq2^R33Q/R33Q^ Mutation Mice

**FIGURE 7:**
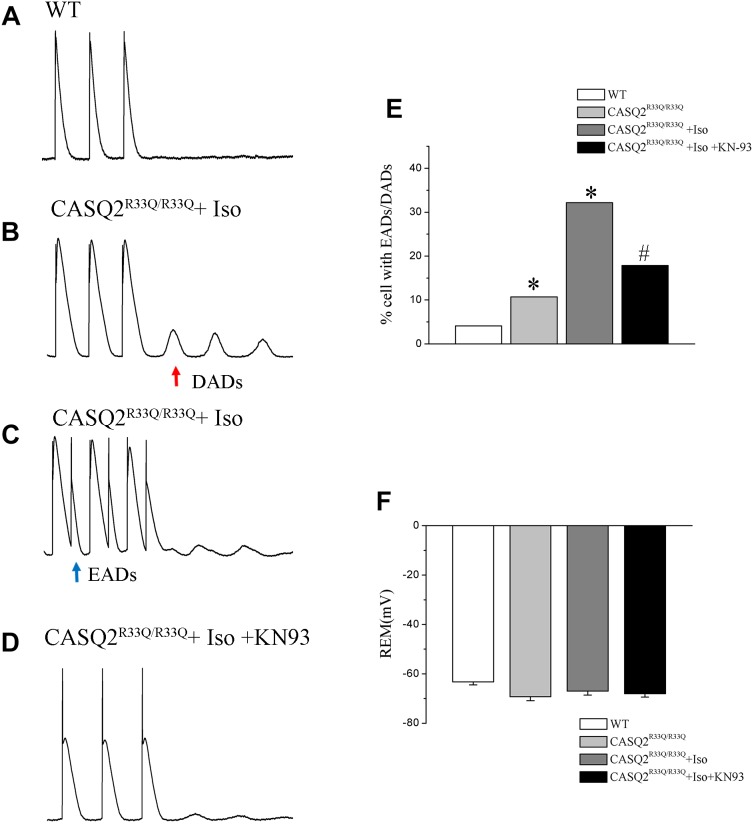
**(A,B)** The occurrence of Ca^2+^ spark in Casq2^R33Q/R33Q^ atrial myocytes with or without Iso, and **(C,D)** the effect of KN92 and KN93 on Casq2^R33Q/R33Q^ atrial myocytes with 1 μM isoproterenol. Comparison of the **(E)** Ca^2+^ spark frequency and **(F)** baseline Ca^2+^ concentration among groups. ^∗^*P* < 0.05 vs. WT, ^∗∗^*P* < 0.01 vs. WT. ^#^*P* < 0.05 when comparing the Iso group with the Iso plus KN92/KN93 group.

All electrical activities were recorded from 28 atrial cells of eight Casq2^R33Q/R33Q^ mice and 24 atrial cells of seven WT mice under 1 nA/2.5 ms pacing. The proportion of atrial cells with oscillation in membrane potentials (EADs or DADs) increased in the Casq2^R33Q/R33Q^ group as compared to the WT group (10.71%, 3/28 vs. 4.16%, 1/24 at 1 Hz, *P <* 0.05). This difference was further promoted by 1 μM Iso in the Casq2^R33Q/R33Q^ group compared with the WT group (32.17 %, 9/28 vs. 12.50%, 3/24 at 1 Hz, *P <* 0.05), and with a significant decrease in the proportion of oscillation in membrane potentials by 1 μM KN93 in the Casq2^R33Q/R33Q^ group (17.85% 5/28 vs. 32.17% 9/28 at 1 Hz, *P <* 0.05). There was no significant difference in the resting membrane potential (RMP) between the two groups with or without Iso (without Iso: -63.21 ± 1.23 mV vs. -69.21 ± 1.66 mV, *P* > 0.05; with Iso: -66.98 ± 1.62 mV vs. -68.03 ± 1.33 mV, *P* < 0.05), indicating that no oscillation in membrane potentials was caused by basal RMP instability (Figure 7).

### The Alternation of Ca^2+^ Transport Channel and Increased CaMKII in the Casq2^R33Q/R33Q^ Atrial Myocytes

**FIGURE 8:**
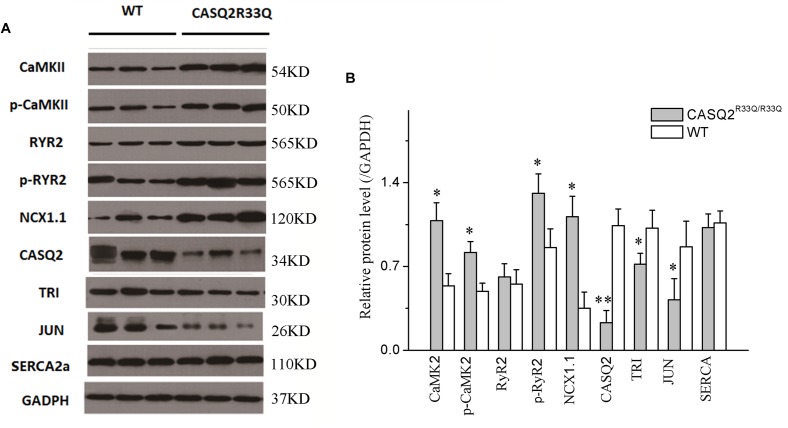
**(A)** Comparison of protein expression between the Casq2^R33Q/R33Q^ mice and the WT mice. **(B)** Quantification of different protein expression shows that CaMKII, p-CaMKII, p-RyR2, and NCX1.1 were promoted, but Casq2, JCT, and TRI were declined in the Casq2^R33Q/R33Q^ atria. CaMKII, calcium/calmodulin-dependent protein kinase II; RyR2, ryanodine receptor; NXC1.1, sodium–calcium exchanger 1.1; Casq2, calsequestrin 2; TRI, triadin; JCT, junctin; SERCA, sarcoplasmic reticulum calcium-ATPases; GAPDH, glyceraldehyde-3-phosphate dehydrogenase. ^∗^*P* < 0.05 vs. WT, ^∗∗^*P* < 0.01 vs. WT.

Western blot was used to compare the levels of protein extracted from the left atria of the Casq2^R33Q/R33Q^ and the WT mice. Compared with the WT group, there were dramatic decreases in the protein levels of Casq2, junctin, and triadin (*n* = 3, *P <* 0.05), but no significant changes in the levels of RyR2 or SERCA (*n* = 3, *P* > 0.05) in the R33Q group. In contrast, there was an increase in the total form of CaMKII and the phosphoryl form of CaMKII or RYR2 (*n* = 3, *P <* 0.05). In addition, atrial NCX1.1 protein expression increased in the R33Q group (*n* = 3, *P <* 0.05) (Figure 8).

## Discussion

In this study, we intended to investigate the effect of Casq2 mutation R33Q on AF. Our major findings are that the Casq2^R33Q/R33Q^ mice were more susceptible to AF, mediated by the increase in SCaEs characterized by Ca^2+^ sparks and Ca^2+^ waves. SCaEs might be responsible for afterdepolarizations (DAD/EAD) via a change in ITi in atrial cells from Casq2^R33Q/R33Q^ mice. CaMKII inhibition reversed the effects of Iso on SCaE and oscillation in membrane potentials and reduced atrial-triggered activity, and hence may be a promising therapeutic strategy for AF.

(1) The increase in the SCaE and the Ca^2+^-sensitive current in Casq2^R33Q/R33Q^ intact atrial cells increases the risk of AF.

Although an SR Ca^2+^leak was found in atrial myocytes of chronic AF patients (Voigt et al., 2012), it is unknown whether the SR Ca^2+^ leak is a cause of chronic AF. Our *in vivo* study demonstrated that Casq2^R33Q/R33Q^ mice with SR calcium channel dysfunction undergo the Ca^2+^ leak from the SR, which increases the basal intracellular Ca^2+^ level, and the Casq2^R33Q/R33Q^ mice have a lower threshold for induction of AF. Consistent with other mouse models with an increased Ca leak, the spontaneous atrial trigger- and pacing-induced AF burden was significantly higher (Faggioni et al., 2014; Guo et al., 2014). Therefore, it is acceptable to consider that the R33Q mutation was enough to induce AF.

Spontaneous Ca^2+^ oscillations have been shown to precede voltage oscillations in multiple studies (Choi et al., 2002; Song et al., 2015). However, it is difficult for Ca^2+^ oscillations alone to produce a self-sustainable action potential (Lou et al., 2015; Song et al., 2015). Therefore, we explored the electrical basis of EADs/DADs by investigating multiple Ca^2+^-dependent ionic currents. Our study demonstrated that late-EADs occur when the potential <-60 mV, and ICa-L is inactivated when the potential <-60 mV. However, ITi can be activated in this range. It is well known that ITi is responsible for forming DADs via the INCX inward current elicited by spontaneous calcium events, and knockout of NCX leads to the reduction of the likelihood that both DADs and EADs will occur (Volders et al., 1997; Pogwizd et al., 2001; Bogeholz et al., 2015). The significant increases in ITi and the incidences of both DADs and EADs corresponded to the increases in Ca^2+^ sparks and Ca^2+^ waves, particularly in Casq2^R33Q/R33Q^ atrial myocytes. Rizzi et al. (2008) found that an abnormal SR Ca^2+^ release is associated with CPVT in CAQS2 mutation mice, emphasizing that diastolic SR Ca^2+^ leak leads to DAD. However, we mainly used our data to study the relationship between the intracellular calcium changes and AF, and EADs also occurred in CAQS2 mutation atrial cells, more than DADs. Therefore, it is likely that SCaE plays a vital role in induced EADs/DADs associated with the development of AF in CAQS2 mutation mice.

(2) The molecular consistency of the RYR2 complex changes for SCaEs in the Casq2^R33Q/R33Q^ mouse.

Previous studies have shown that the SR calcium channel receptor RYR2 and RyR2-stable subunits (including Casq2, triadin, and junctin) are involved in Ca^2+^ release (Beavers et al., 2013). A reduction of Casq2 has been observed in aging patients with AF (Herraiz-Martinez et al., 2015). Moreover, Casq2 reduction has also been observed in the atrial myocytes of congestive heart failure patients that are associated with risk of AF (McFarland et al., 2010). It is possible that the acquired Casq2 reduction might promote AF. Triadin and junctin act as Casq2 anchor proteins that mediate Casq2-RYR2 interactions (Franzini-Armstrong, 2009; Liu et al., 2009). Franzini-Armstrong (2009) discovered that a change in the amounts of Casq, RYR2, triadin, and junctin induced different structures of the RYR2 complex, indicating that the proportion of these proteins is important for the function of the RYR2 complex. Casq2 mutations alter the sensitivity of RYR2 Ca^2+^ release, but it remains to be clarified whether the molecular ratio between Casq2 and the three proteins is the key point for the abnormal structure of SR calcium release units in Casq2^R33Q/R33Q^ mutant atrial cells.

The animal model in this study elucidates the abnormal SR membrane calcium channels responsible for abnormal calcium leak or release from the SR. The remodeling of Ca^2+^-handling proteins has been considered as the molecular basis for the onset of AF (Voigt et al., 2014). Our study demonstrated that the levels of triadin and junctin were significantly decreased in the R33Q mutant atrial tissue, but the RYR2 expression levels were similar between the R33Q mutant and the WT groups, suggesting that a functional change likely occurred in the RYR2 receptor rather than a significant change in the number of receptors in the R33Q mutant atrial myocytes. The dysfunction of RYR2 due to reduced RYR2-stable subunits results in instability characterized by calcium leaking or releasing (Hake et al., 2012). A similar change was also observed in ventricular cells from Casq2^R33Q/R33Q^ mice with ventricular tachycardia (Rizzi et al., 2008). Therefore, the dysfunction of the SR membrane calcium channel receptor RYR2 secondary to reduced RyR2-stable subunits may be associated with the development of AF or ventricular tachycardia through the common pathway via SR Ca^2+^ leakage and abnormal release-triggered activity.

(3) Disordered Ca^2+^ dynamic by Iso and CaMKII-mediated RyR2 phosphorylation as pro-arrhythmic factors in Casq2^R33Q/R33Q^ atrial cells.

β-Adrenergic activity has been proven to trigger ventricular arrhythmias via catecholaminergic polymorphic ventricular tachycardia (Sumitomo et al., 2007; Shan et al., 2012). An acute exposure of adult atrial myocytes to Iso significantly shortens the decay time of Ca^2+^ release and the time to Ca^2+^ peak. Shortening of the decay time of Ca^2+^ release indicates an increased SERCA2a function (Gao et al., 2017). However, the SERCA2a protein expression was not affected by R33Q mutation. A possible reason for this is that a change in the Ca^2+^ leak from the SR slows the rate of decay of the Ca^2+^ transient (Belevych et al., 2007; Guo et al., 2012; Sankaranarayanan et al., 2016). Shortening the time to Ca^2+^ peak suggests an increase in the sensitivity of the calcium channel RyR2 (Li et al., 1998) that enhances RyR2-induced Ca^2+^ release. The increases in the RyR2 channel sensitivity will lead to abnormal Ca^2+^ handling and intracellular Ca^2+^ accumulation, and to the increase in calcium-sensitive calcium leakage, thus prolonging the rate of decay of the Ca transient. Our study demonstrated that Iso decreased the decay time of Ca^2+^ release and the time to Ca^2+^ peak, and increased SCaE, ITi, and the incidences of EADs/DADs in atrial cells from the Casq2^R33Q/R33Q^ mice, suggesting that Ca^2+^ triggered the activity for initiating AF in the R33Q mice.

Calmodulin-dependent protein kinase II overactivity is an important pro-arrhythmic factors in several cardiac pathologies. We found that CaMKII-dependent RyR2 phosphorylation (Ser 2814) is enhanced in R33Q hearts. Popescu et al. (2016) showed that the increased incidence of pro-arrhythmogenic Ca^2+^ sparks and waves is caused by CaMKII activity and CaMKII-mediated RyR phosphorylation in AnkB^+/-^ hearts. Terentyev et al. (2014) demonstrated that RyR-mediated Ca^2+^ releases are caused by RyR hyperphosphorylation in long QT syndrome-2, underlying the enhanced triggered activity. Moreover, blocking CaMKII alone reduces RyR2 sensitivity with an effect on Ca^2+^ leaking and ITi density, particularly in the situation of β-adrenergic stimulation. These findings further indicated that the CaMKII-mediated RyR2 phosphorylation may underlie the mechanism of arrhythmia in the R33Q atrium and showed the therapeutic strategy for the prevention of AF by the inhibition of CaMKII and the regulation of Ca^2+^ disorder.

### Limitations of This Study

Although a high susceptibility to AF was demonstrated in this *in vivo* study of Casq2^R33Q/R33Q^ mice, most other results were drawn from *in vitro* studies. It is unknown whether these changes in Ca^2+^, ITi, and oscillation in membrane potentials play the same roles in the development of AF in live animals or humans. We were unable to separately investigate EADs and DADs or their different roles in the development of AF due to the constant occurrence of EADs with DAD in our study. We were also unable to study outward currents during phases 3 and 4 of the action potential. Our future direction is to further clarify the individual role of Ca^2+^-induced EADs and DADs in the development of AF.

## Ethics Statement

The investigation complied with the Guide for the Care and Use of Laboratory Animals published by the US National Institutes of Health (NIH Publication No. 85-23 revised 1996).

## Author Contributions

J-cZ and H-lW conceived and designed the research, performed the acquisition, analyses, and interpretation of the data, obtained funding, supervised the work, drafted the manuscript, and critically revised the manuscript for important intellectual content. QC, X-tX, TZ, and G-jX performed the acquisition, analyses, and interpretation of the data, and drafted the manuscript. YD, CZ, and LY drafted the manuscript and critically revised the manuscript for important intellectual content. YL and P-lZ conceived and designed the research, obtained funding, supervised the work, and critically revised the manuscript for important intellectual content.

## Conflict of Interest Statement

The authors declare that the research was conducted in the absence of any commercial or financial relationships that could be construed as a potential conflict of interest.
